# Impact of pericardial adhesions on diastolic function as assessed by vortex formation time, a parameter of transmitral flow efficiency

**DOI:** 10.1186/1476-7120-8-42

**Published:** 2010-09-22

**Authors:** Panupong Jiamsripong, Mohsen S Alharthi, Anna M Calleja, Eileen M McMahon, Minako Katayama, John Westerdale, Michele Milano, Jeffrey J Heys, Farouk Mookadam, Marek Belohlavek

**Affiliations:** 1 Translational Ultrasound Research Laboratory, Division of Cardiovascular Diseases, Mayo Clinic, Scottsdale, Arizona, USA; 2 Department of Mechanical Engineering, Arizona State University, Tempe, AZ, USA; 3 Chemical and Biological Engineering, Montana State University, Bozeman, MT, USA

## Abstract

**Background:**

Pericardial adhesions are a pathophysiological marker of constrictive pericarditis (CP), which impairs cardiac filling by limiting the total cardiac volume compliance and diastolic filling function. We studied diastolic transmitral flow efficiency as a new parameter of filling function in a pericardial adhesion animal model. We hypothesized that vortex formation time (VFT), an index of optimal efficient diastolic transmitral flow, is altered by patchy pericardial-epicardial adhesions.

**Methods:**

In 8 open-chest pigs, the heart was exposed while preserving the pericardium. We experimentally simulated early pericardial constriction and patchy adhesions by instilling instant glue into the pericardial space and using pericardial-epicardial stitches. We studied left ventricular (LV) function and characterized intraventricular blood flow with conventional and Doppler echocardiography at baseline and following the experimental intervention.

**Results:**

Significant decreases in end-diastolic volume, ejection fraction, stroke volume, and late diastolic filling velocity reflected the effects of the pericardial adhesions. The mean VFT value decreased from 3.61 ± 0.47 to 2.26 ± 0.45 (P = 0.0002). Hemodynamic variables indicated the inhibiting effect of pericardial adhesion on both contraction (decrease in systolic blood pressure and +dP/dt decreased) and relaxation (decrease in the magnitude of -dP/dt and prolongation of Tau) function.

**Conclusion:**

Patchy pericardial adhesions not only negatively impact LV mechanical functioning but the decrease of VFT from normal to suboptimal value suggests impairment of transmitral flow efficiency.

## Background

Left ventricular (LV) diastolic filling and its alterations are important indicators of overall cardiac health status [1-4] and contribute to early diagnosis of cardiovascular disease [1,5]. We have shown by echocardiographic particle imaging velocimetry [6], and other investigators have demonstrated by magnetic resonance imaging [7], that transmitral flow can produce an intraventricular rotational body of fluid referred to as a vortex ring [8-10]. This vortex supports more efficient fluid transport as compared to a straight jet alone [11,12]. Gharib et al. [13] derived a dimensionless index that quantitatively characterizes optimal fluid dynamic conditions for vortex formation from the duration of flow through an orifice with known diameter and, thus, referred to the index as vortex formation time (VFT). VFT measurement, when applied to diastolic filling, suggests an efficient diastolic transmitral flow if the resulting value is within an optimal range from 3.5 to 4.5 [13]. We have demonstrated that when LV filling is altered, such as during increased LV afterload, the resulting mean VFT value in fact shifts out of the optimal range [14].

Development of pericardial adhesions is a pathophysiological marker of constrictive pericarditis (CP), which impairs cardiac filling by limiting the total cardiac volume compliance [15,16]. The current study focuses on the diastolic filling and VFT parameters and its alteration in an animal model of pericardial adhesions [17]. Capitalizing on existing data from our previous studies we hypothesized that experimentally induced pericardial adhesions will be hemodynamically accompanied not only by impairment in conventional Doppler flow characteristics but also by decreased transmitral flow efficiency, as assessed by VFT.

## Materials and methods

### Animal Preparation

The study was approved by the Mayo Clinic Institutional Animal Care and Use Committee. Eleven pigs weighing 40 to 45 kg were anesthetized with inhalation of 1.5% isoflurane and mechanically ventilated with a mechanical ventilator (Ohmeda 7800 Ventilator; Datex-Ohmeda Inc, Madison, Wisconsin), and blood gases were periodically checked. Pressures in the LV and ascending aorta were measured with high-fidelity catheters (Millar Instruments, Houston, TX) placed via internal carotid artery cannulation. Internal jugular vein cannulation allowed administration of fluids and medications. After median sternotomy, the heart was exposed, sparing the pericardium.

### Experimental Intervention

**Figure 1:**
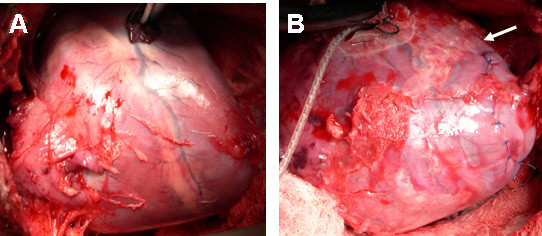
**A) Heart with normal pericardium at baseline; B) Heart with stitched and glued pericardium.** The arrow points at visible adhesions.

We have described the pericardial adhesion model in detail previously [17] (Figure 1). Briefly, pericardial-epicardial adhesions were induced with an ethyl-cyanoacrylate glue (Super Glue™; Pacer Technology, LLC, Rancho Cucamonga, California). Cyanoacrylate instillation induced patchy adhesions and a thickened shell that encompassed approximately 70% of the LV pericardial surface.

### Hemodynamic Data Analysis

LV end-diastolic pressure (LVEDP) was timed to the R-wave peak on the synchronous electrocardiographic (ECG) tracing. Besides peak positive dP/dt (+dP/dt) and peak negative dP/dt (-dP/dt), the time constant of LV pressure decay during the isovolumic relaxation period (Tau) was ascertained by using a validated zero-asymptote model [18].

### Echocardiographic Image Acquisition

An Acuson Sequoia C512 ultrasound system (Siemens Medical Solutions Inc, Mountain View, California) equipped with an 8V3C transducer set to 3.5 MHz was used to acquire echocardiographic images. The transducer was placed on the pericardium and acoustically coupled with a small amount of gel. Ultrasound scans for each pig included: 1) apical 2-chamber and 4-chamber views for measurements of LV cardiac output, end-diastolic volume (EDV), end-systolic volume, stroke volume, and ejection fraction; 2) Doppler spectral flow for peak early (E-wave) and atrial (A-wave) filling velocities; and 3) tissue Doppler for peak velocities of the mitral annulus in early (E') phases of LV filling (measured at the basal medial portion of the mitral annulus), and the dimensionless ratio E/E' was computed [19,20]. For each scan, 3 cardiac cycles were acquired at a frame rate of 60-70 Hz.

### VFT Definition

Transmitral flow can produce an intraventricular rotating fluid mass referred to as a vortex ring which supports a more efficient fluid transport compared to a straight jet alone [11,12]. The vortex ring minimizes energy dissipation during fluid ejection through an orifice [21] and optimizes to facilitate the efficiency of fluid transport. The formation of abnormal vortices also relates to the underlying fluid dynamics in LV dysfunction and abnormal LV loading condition [13,22]. Therefore, vortex flow may offer a novel index for blood transport efficiency and may be of incremental value as a marker of cardiac disease. Gharib and colleagues [13] derived the VFT formula, which we refer to as "conventional". The formula is based on LV function, mitral orifice diameter, and diastolic filling flow velocities, ie, parameters that can be measured noninvasively by echocardiography:

$$\text{VFT}=\frac{4\;(1-\beta )\;\alpha^{3}}{\pi }\times \text{EF},$$

where β is the fraction of the stroke volume provided by the atrial component of LV filling and was calculated as the A-wave area divided by the sum of the A-wave and E-wave areas [13]. In our study, we estimated β by using the peak magnitude of the A-wave divided by the sum of the A-wave and E-wave peak magnitudes. The symbol α represents an LV geometry parameter defined as $\alpha =\frac{{\text{EDV}}^{1/3}}{\text{D}}$. The α parameter incorporates measurements of EDV in mL and mitral orifice diameter (D) in cm. The latter has been obtained in our study by averaging the largest mitral orifice diameters obtained during early diastolic filling in the 4-chamber and 2- or 3-chamber apical views.

However, vortex ring formation during diastole can also be defined as [22]:

$$\text{VFT}=\frac{\bar{{\text{U}}_{\text{t}}}}{\text{D}}\times \text{T,}$$

where $\bar{{\text{U}}_{\text{t}}}$ (cm/s) is time-averaged velocity of the fluid flow, T is the duration of fluid ejection, and D (cm) is the diameter of the mitral annulus obtained in our study as the mean of the largest diameters measured during early diastolic filling in the 2, 3, or 4-chamber apical views [13]. Because the vortex ring of interest forms in the early phase of diastolic filling, the term $\bar{{\text{U}}_{\text{t}}}\times \text{t}$ can be measured echocardiographically using the time-velocity integral of the E-wave, ie, TVI_E _(cm). This leads to a simple definition of vortex formation time (ie, VFTs), which has been used in our study as follows:

$$\text{VFTs}=\frac{{\text{TVI}}_{\text{E}}}{\text{D}}.$$

We used this formula because of its practicality and we believe that this formula is a more convenient and more practical implication.

### Data Analyses

Data were expressed as means ± standard deviation. Hemodynamic, 2 D, and Doppler echocardiographic data, as well as strain and strain rate (SR) measurements were obtained at baseline and during intervention. These data were compared using a 2-tailed paired t test. The relationship between different continuous variables was analyzed using single and multiple regression analyses. All differences were considered significant for P < 0.05.

## Results

Data were obtained from 8 animals and were used to induce the pericardial adhesion model [14] and generate the results of this study.

### Hemodynamic Parameters

Hemodynamic results are summarized in Table 1. Heart rate did not change. Systolic blood pressure decreased after intervention, while diastolic blood pressure and LV end-diastolic pressure did not significantly change. However, magnitudes of +dP/dt and -dP/dt decreased significantly while Tau has been prolonged, suggesting an inhibiting effect of the experimental pericardial adhesions on the rate of LV contraction and relaxation. These changes coincided with a decrease in cardiac output. We did not observe respiratory variations or the characteristic dip and plateau of the LV pressure tracing.

**Table 1:** Hemodynamic Parameters

Variable	Baseline	Adhesions	*P *value
BPs (mm Hg)	111.59 ± 10.55	94.66 ± 20.51	0.0382
BPd (mm Hg)	78.10 ± 7.70	66.54 ± 21.91	0.1276
BPm (mm Hg)	89.26 ± 8.37	75.91 ± 21.33	0.0842
HR (beats/min)	77.50 ± 7.91	84.88 ± 9.43	0.2230
CO (mL/min)	2.66 ± 0.90	1.44 ± 0.48	0.0005
LVEDP (mm Hg)	7.50 ± 3.78	9.29 ± 4.76	0.4239
+dP/dt (mm Hg/sec)	1096.50 ± 236.41	795.50 ± 155.03	0.0005
-dP/dt (mm Hg/sec)	-1490.63 ± 198.60	-1044.75 ± 219.31	0.0002
Tau (msec)	42.46 ± 9.06	70.66 ± 34.81	0.0470

BPd, blood pressure diastolic; BPm, mean arterial blood pressure; BPs, blood pressure systolic; CO, cardiac output; +dP/dt, maximum positive LV pressure change; -dP/dt, maximum negative LV pressure change; HR, heart rate; LVEDP, left ventricular end-diastolic pressure; Tau, the time constant of isovolumic relaxation.

### Echocardiographic Parameters

Conventional echocardiographic measurements of LV filling and mechanical function are summarized in Table 2. Peak A-wave (but not E-wave) velocity decreased and the E/A ratio showed an increasing trend, suggesting impairment in LV preload. E/E' did not change. While end-systolic volume did not change either, EDV decreased significantly, resulting in a post-intervention drop in cardiac output, stroke volume, and ejection fraction.

**Table 2:** Echocardiographic Parameters

Variable	Baseline	Intervention	*P *value
E (m/s)	0.49 ± 0.16	0.44 ± 0.04	0.3228
A (m/s)	0.55 ± 0.12	0.42 ± 0.13	0.0342
E/A	0.92 ± 0.28	1.12 ± 0.32	0.1162
E/E'	5.21 ± 1.60	4.03 ± 1.15	0.1310
EDV (mL)	60.73 ± 18.11	42.30 ± 11.13	0.0008
ESV (mL)	25.50 ± 7.65	24.31 ± 6.83	0.5223
SV (mL)	34.49 ± 10.55	17.22 ± 4.95	0.0002
EF (%)	57.03 ± 2.44	40.66 ± 6.20	0.0001

A, peak transmitral flow velocities at late filling phases; E, peak transmitral flow velocities at early filling phase; E', early diastolic myocardial peak velocity septal annulus; EDV, left ventricular end-diastolic volume; EF, ejection fraction; ESV, left ventricular end-systolic volume; SV, stroke volume.

### Vortex Formation Parameters

**Figure 2:**
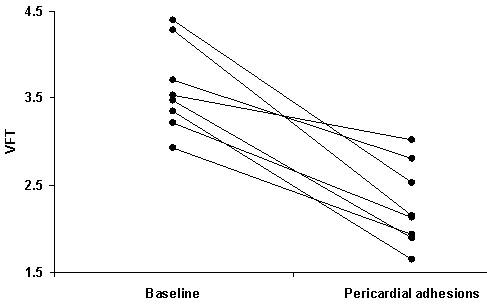
**Change of vortex formation time (VFT) value from baseline to intervention**.

The mean values of D, when compared between baseline and intervention, remained nearly identical (Table 3). TVI_E _decreased and resulted in a significant decrease in VFT after the intervention (Figure 2).

**Table 3:** Vortex Formation Parameters

Variable	Baseline	Intervention	*P *value
D (cm)	1.76 ± 0.18	1.78 ± 0.15	0.7660
TVI_E_	6.31 ± 0.99	4.05 ± 0.85	0.0004
VFT	3.61 ± 0.47	2.26 ± 0.45	0.0002

D, diameter of mitral valve orifice; TVI_E_, time-velocity integral of the E-wave; VFT, vortex formation time.

## Discussion

To our knowledge, this study shows for the first time that experimentally simulated early (patchy) formation of a noncompliant pericardium with adhesions negatively affects transmitral flow efficiency, as expressed by a shift in VFT values out of their optimal range.

### Model of Pericardial Thickening and Adhesions

Our animal model produced areas of pliable and rigid pericardium (based on direct palpation during the open-chest study) and strong pericardial-epicardial adhesions. In a fully developed model of CP, the encasement of the heart by a rigid shell of noncompliant pericardium results in characteristic pathophysiologic effects, including impaired diastolic filling of the ventricles [23].

Hemodynamic parameters (Table 1) and functional parameters (Table 2) were significantly affected by the experimentally induced pericardial adhesions. Septal bounce was seen in 5 out of 8 animals. However, respiratory variation of the E- wave was not consistently present nor was a high E/A ratio. The lack of these diagnostic characteristics was likely due to controlled respiration in the sedated and mechanically-ventilated animals.

### LV Systolic and Diastolic Function Following Induction of Pericardial Adhesions

In our model, LV function was impaired in its mechanical performance by patchy pericardial-epicardial adhesions and spotty stiffening or hardening of the pericardium led to impairment of both systolic and diastolic myocardial function. The observed decreases in ejection fraction, stroke volume, cardiac output, and +dP/dt are consistent with impairment in systolic function, but the mechanism of reduced ejection performance may be secondary to impaired ventricular filling, especially in mid- and late diastole (reduced preload) [15].

The decrease in the absolute value of -dP/dt and an increase in Tau are consistent with impairment in diastolic function. Impairments in LV filling (due to pericardial constriction) [24,25] are the most likely mechanisms of diastolic dysfunction. The impairment of LV filling typically presents at early diastole by a high E velocity and E/A ratio. But due to the acute study, some conventional echocardiographic findings, such as a high E/A ratio and significant transmitral or tricuspid E-wave respiratory variations, were absent.

Although the possibility of injury and its effect on LV function cannot be excluded, Kaplan et al [26] showed that ethyl-cyanoacrylate tissue glue does not cause foreign body, inflammatory, necrotic, or other histopathologic reactions when used for bonding of cardiovascular tissues. Also, the pericardial-epicardial stitches were placed very carefully, with no subsequent bleeding or apparent injury.

### Changes in VFT and its Component Parameters with Pericardial Adhesions

LV diastolic filling plays an important role in cardiac function and its optimal means of cardiac efficiency [13]. LV diastolic function can be assessed by invasive and non-invasive methods. Doppler flow echocardiography is the most practical routine clinical approach of a non-invasive method for evaluating LV diastolic function. Doppler is performed to obtain mitral inflow velocities during diastole which has peak E (early diastolic) and A (late diastolic) [27]. E wave is predominant LV filling from the LA to the LV. The flow of blood during E wave occurs from the rapid pressure drop in LV by accelerating through the mitral valve and has been observed to induce formation of a rotating fluid of blood called a vortex ring [13].

The diastolic vortex ring acts as a kinetic energy reservoir that facilitates propulsion of blood in systole [21], contributes to blood redirection into the outflow tract and aorta [28,29], and helps in preventing blood stagnation within the LV apex [29]. Based on mathematical modeling, the vortex ring may exert a force on the mitral valve, thus contributing to its timely closure [30]. Premature disintegration of the vortex and dissipation of the stored energy eliminates the beneficial effects of vortex formation and may necessitate an increase in the physical work generated by the cardiac muscle, thus augmenting the oxygen consumption, which in turn reduces the efficiency of the heart [31]. The process of vortex ring formation during diastole can serve as an indicator of cardiac health [13].

The rapid development of the adhesions in our study impairs both systolic and diastolic myocardial function. Consequently, as our study shows, this alteration also negatively impacts conditions for vortex ring formation, (indicated by the mean VFT value shift to below the optimal range) and implies the loss of optimal hemodynamic efficiency during LV filling [32].

### Clinical Implications

This is a starting point towards further investigations of VFT in chronic pericarditis both in animal experiments and clinical practice. Diagnosis of early stages of CP can be challenging because of variable hemodynamic presentations and the difficulty of distinguishing CP from other diseases with similar symptoms and signs [33]. VFT may supersede conventional parameters of cardiac mechanical or hemodynamic function, because it is a disease stage independent factor as well as a patient effects independent parameter [13]. VFT is a dimensionless index that is easy to perform with simple echocardiographic principles, it is a dimensionless index defined on a universal timescale [22] and hence has practical advantages. As we have shown, our animal model replicates pericardial adhesion and the VFT parameter contributes to a conceptually novel interpretation of cardiac function (ie, efficiency of diastolic blood transport) in such a situation.

### Limitations

Spreading the tissue glue within the pericardial space was a manual process, which introduced variations in the distribution of adhesions and pericardial stiffening. However, despite the variability in this process, we observed a consistent and statistically significant decrease in VFT. We cannot conclude that this animal model of pericardial adhesion is the same as physiology in chronic pericarditis. We plan on conducting further studies, both acute and chronic, to continue the development of the animal model and to characterize its functional and histopathologic effects on myocardium. There is a slight inconsistency between the optimal VFT value range considered for experimental in vitro setting and clinical settings; the latter has a somewhat broader range [13]. Thus, VFT needs to be broadly investigated in normal and disease conditions both in the clinical and experimental animal setting.

## Conclusion

In our animal model, patchy pericardial stiffening and pericardial-epicardial adhesions resulted in systolic and diastolic dysfunction. However besides the mechanical effect, we found also detrimental effect of pericardial adhesion on diastolic vortex function, a decrease in the VFT value, suggesting impairment in the efficiency of diastolic filling.

## Competing interests

The authors declare that they have no competing interests.

## Authors' contributions

JP conceived the study and participated in the design and creation, performed detailed statistical analyses, and drafted manuscript (original and revision). MA critically reviewed study and manuscript. AC critically reviewed study and manuscript. EM critically reviewed study and manuscript. MK critically reviewed the manuscript. JW critically reviewed VFT analyses. MM critically reviewed VFT analyses. JH critically reviewed VFT analyses. FM critical review of manuscript. MB participated in design and creation of study and critically reviewed statistical analyses and manuscript. All authors read and approved the final manuscript.
